# Structural Augmentation in Rotator Cuff Repair Decreases the Risk of Retear: A Systematic Review and Meta-analysis

**DOI:** 10.1177/03635465251400356

**Published:** 2026-01-18

**Authors:** Danielle Dagher, Darshil Shah, Colin Kruse, Hassaan Abdel Khalik, Usama Saleh, Abdul-Ilah Hachem, John M. Tokish, Asheesh Bedi, Moin Khan

**Affiliations:** * Department of Health Research Methods, Evidence, and Impact, McMaster University, Hamilton, Ontario, Canada; † Division of Orthopaedic Surgery, Department of Surgery, McMaster University, Hamilton, Ontario, Canada; ‡ Medcare Orthopaedics and Spine Hospital, Dubai, United Arab Emirates; § Department of Orthopedic Surgery and Traumatology, University Hospital Bellvitge, Barcelona, Spain; ‖ Department of Orthopedic Surgery, Mayo Clinic, Scottsdale, Arizona, USA; ¶ Department of Orthopaedic Surgery, University of Michigan, Ann Arbor, Michigan, USA; Investigation performed at McMaster University, Hamilton, Ontario, Canada

**Keywords:** rotator cuff, augmentation, allograft, xenograft, autograft, systematic review

## Abstract

**Background:**

Rotator cuff tears are a common orthopaedic injury often resulting in shoulder pain and functional impairment. Despite the prevalence of rotator cuff tears, the incidence of retear after rotator cuff repair (RCR) remains high. There is growing interest in the use of structural augmentation in RCR to decrease retear rates and improve patient outcomes.

**Purpose:**

The purpose of this systematic review and meta-analysis was to compare the clinical and imaging outcomes of structural augmentation versus standard RCR in patients with full-thickness rotator cuff tears. The effectiveness of different augment subtypes was also assessed.

**Study Design:**

Meta-analysis; Level of evidence, 3.

**Methods:**

A comprehensive literature search was conducted in CENTRAL, Embase, and Medline from inception to November 13, 2024. Eligible studies included comparative trials evaluating structural augmentation in RCR for repairable full-thickness tears. Risk of bias was assessed using the Cochrane risk-of-bias tool for randomized controlled trials and the MINORS (Methodological Index for Non-randomized Studies) score for observational studies. Meta-analyses were performed via a random effects model, with subgroup analyses performed by study design and augment type.

**Results:**

Twenty-four studies (12 randomized controlled trials and 12 cohort studies) were included in this review, all of which evaluated biologic augments (xenograft, allograft, autograft). Structural augmentation significantly reduced the risk of retear by 35% as compared with standard RCR (risk ratio, 0.65; 95% CI, 0.48-0.89; *P* = .01). Retears were confirmed radiographically by magnetic resonance imaging or ultrasound. Subgroup analysis revealed the greatest reduction in retear rates with allograft augmentation (risk ratio, 0.34; 95% CI, 0.21-0.56). No significant differences were observed in patient-reported outcomes, including pain scores, American Shoulder and Elbow Surgeons scores, or Constant scores.

**Conclusion:**

Structural augmentation in RCR significantly reduces retear rates, particularly with allografts, but does not improve short-term patient-reported outcomes. Further research is needed to identify which patient and tear characteristics may influence the effectiveness of augmentation.

A rotator cuff tear is a debilitating condition that commonly results in shoulder pain and functional impairment. While some tears can be effectively managed through nonoperative treatment, surgical intervention may be necessary for others with refractory pain, weakness, or reduced active range of motion.^41^ The incidence of retear after surgery has been reported to be between 10% and 60%.^18^ Although the occurrence of a structural retear does not immediately correlate with functional deficits, it is associated with a decline in patient-reported outcomes (PROs) over the long term.^36^ The economic impact of rotator cuff repair (RCR) failure is substantial, with estimates suggesting a financial burden >$438 million in the United States within 2 years after surgery.^83^ Over 50% of this cost is estimated to be due to nonoperative management of these retears.

Despite a significant increase in RCRs over the past 2 decades, improvements in retear rates have been minimal.^54^ Several studies have explored factors influencing rotator cuff retears.^9,15,19,27,28,32,50^ These studies have found that addressing modifiable patient-related factors—such as nutritional status, diabetes and hyperlipidemia management, and bone density enhancement through parathyroid hormone—can improve healing prospects.^58,68^ However, nonmodifiable factors such as age, tear characteristics, and revision surgery continue to significantly affect healing outcomes.^27,55,61,74^ Older age (>55 years) and anterior L-shaped tears have been particularly associated with higher retear rates, and revision surgery nearly doubles the risk of retear.^9,66,67^ Enhancing tendon healing in the presence of these nonmodifiable risk factors remains a challenge. Predictive indices have been developed to estimate retear risk, highlighting that patients with an anteroposterior tear size ≥4 cm, a critical shoulder angle >37°, and hyperlipidemia face an 86% likelihood of retear.^31,39,42^ These indices may help inform surgical decisions, guiding tailored interventions for patients at high risk. Although advancements in repair techniques such as double-row fixation, transosseous equivalent, and interval slides have positively affected healing rates, the retear rate remains persistently high.^44,56,75^

Augmentation of RCRs has been proposed to enhance biological healing. This can be achieved through cellular injections or structural scaffolds. Cellular methods include mesenchymal stem cells, platelet-rich plasma, bone marrow stimulation, and bone marrow aspirate concentrate, although their efficacy remains debated.^23,77^ There has been growing interest in the use of structural augments to improve the healing environment.^25^ Structural augments can be broadly categorized as synthetic or biologic, with the latter divided into autografts, allografts, and xenografts.^70^ Structurally, augments may serve as interposition or onlay grafts. Interposition grafts bridge the gap between native tissue and the humeral tuberosity, while onlay grafts are typically placed over the repaired tissue. In some cases, augments are positioned between the rotator cuff and humeral tuberosity.^7,62^

Despite the growing interest in using structural augments in RCR, evidence supporting their effectiveness remains scarce. Existing studies are limited by small sample sizes, making it difficult for clinicians to make evidence-based decisions regarding augment use in RCR. This highlights the need for a comprehensive synthesis of the available literature. The purpose of this systematic review and meta-analysis was to compare the clinical and imaging outcomes of structural augmentation versus standard RCR. Additionally, the review analyzed the comparative effectiveness of different augment types, offering insights to guide future research.

## Methods

### Eligibility Criteria

This review included studies comparing standard RCR versus RCR with structural augmentation in patients with full-thickness rotator cuff tears. Primary and revision cases were included. The following were excluded: partial-thickness tears, irreparable tears, nonstructural augmentations (ie, injections), interposition grafts, noncomparative studies, animal studies, cadaver studies, review articles, and technique articles.

### Search Strategy and Screening

This review was performed according to the methods outlined in chapter 4 of the *Cochrane Handbook for Systematic Reviews of Interventions* and reported according to PRISMA guidelines.^47^ CENTRAL (Cochrane), Embase (OVID), and Medline (OVID) were searched from inception to November 13, 2024. Search strategies were tailored per database (see Appendix 1, available in the online version of this article). Two reviewers (D.D. and D.S.) independently screened studies for potential eligibility based on titles and abstracts in the Covidence systematic review software (Veritas Health Innovation). The same reviewers independently conducted full-text screening, and any disagreements were resolved through consensus. Interrater reliability was measured at the title and abstract stage as well as the full-text screening stage using Cohen kappa (κ) coefficients. Agreement was categorized a priori per Landis and Koch, with a value of 0.81 to 1.0 representing near perfect agreement, 0.61 to 0.80 substantial agreement, 0.41 to 0.60 moderate agreement, 0.21 to 0.40 fair agreement, and 0.00 to 0.20 slight agreement.^43^

### Data Abstraction

Two reviewers (D.D. and D.S.) independently extracted data from the studies into data extraction sheets designed a priori on Microsoft Excel. Extracted data included study characteristics (title, author, study design, eligibility criteria, sample size, patient demographics), details regarding the interventions and comparators (augment type, repair technique, concomitant procedures), and results of the outcomes (imaging and patient reported). Any discrepancies were resolved through discussion among reviewers.

### Quality Assessment

Two reviewers (D.D. and D.S.) independently assessed the quality of the studies. For RCTs, the risk of bias was assessed by the revised Cochrane risk-of-bias tool for randomized trials (RoB-2).^33^ The following methodological domains were assessed: randomization process, deviations from the intended interventions (effect of assignment to intervention), missing outcome data, measurement of the outcome, and selection of the reported result. Each domain was rated as “low risk,”“high risk,” or “some concerns” with a study-level risk-of-bias judgment being given according to the criteria provided by the RoB-2 tool. Disagreements were resolved by consensus between the reviewers. For observational studies, the quality was assessed per the MINORS (Methodological Index for Non-randomized Studies) score.^69^ This tool consists of 12 items for comparative studies, with each item given a rating of 0 (not reported), 1 (reported and inadequate), or 2 (reported and adequate), for a maximum score of 24 points. For the purposes of this review, a score ≤14 was considered poor quality, 15 to 19 moderate quality, and 20 to 24 good quality.

### Assessment of Heterogeneity

Clinical and methodological heterogeneity was assessed by examining study and participant characteristics. Statistical heterogeneity was assessed with the *I*^2^ statistic. The interpretation of the *I*^2^ statistic was based on the following guidelines from the Cochrane handbook: 0% to 40% might not be important, 30% to 60% may represent moderate heterogeneity, 50% to 90% substantial heterogeneity, and 75% to 100% considerable heterogeneity.^24^

### Statistical Analyses

Pairwise evidence was meta-analyzed in DataParty. Data were pooled per the random effects model based on the assumption that clinical heterogeneity was likely to exist and have an effect on the results. In cases where trials evaluated multiple intervention arms against a single comparator, the groups were analyzed separately. To avoid double counting, the sample size for the comparator group was divided by the number of times that the group was included in the analysis. For the binary outcome (retears), risk ratio and 95% confidence interval (95% CI) were calculated. All continuous outcomes were measured via comparable methods and thus analyzed by a mean difference (MD) and 95% CIs. For outcomes assessed at multiple time points, results at 12 months were utilized in the analyses. In studies that did not report results at 12 months, the closest time point to 12 months was used. Subgroup analyses were performed for all meta-analyzed outcomes based on study design and augment type.

## Results

### Characteristics of Included Studies

**Figure 1.**
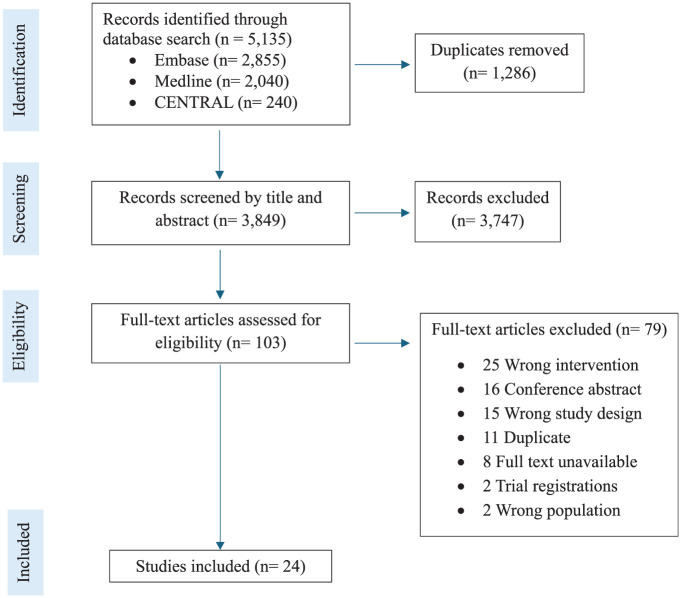
PRISMA flow diagram.

The initial search across 3 databases identified 5135 records, of which 3849 remained after duplicate removal. After screening by title and abstract, 103 studies remained for full-text assessment of eligibility, of which 24 were included in this review (Figure 1).^**^ Interrater reliability, measured using Cohen κ coefficients, yielded values of 0.727 and 0.798 at the title/abstract and full-text stages, respectively, with both values representing substantial agreement.

**Figure 2.**
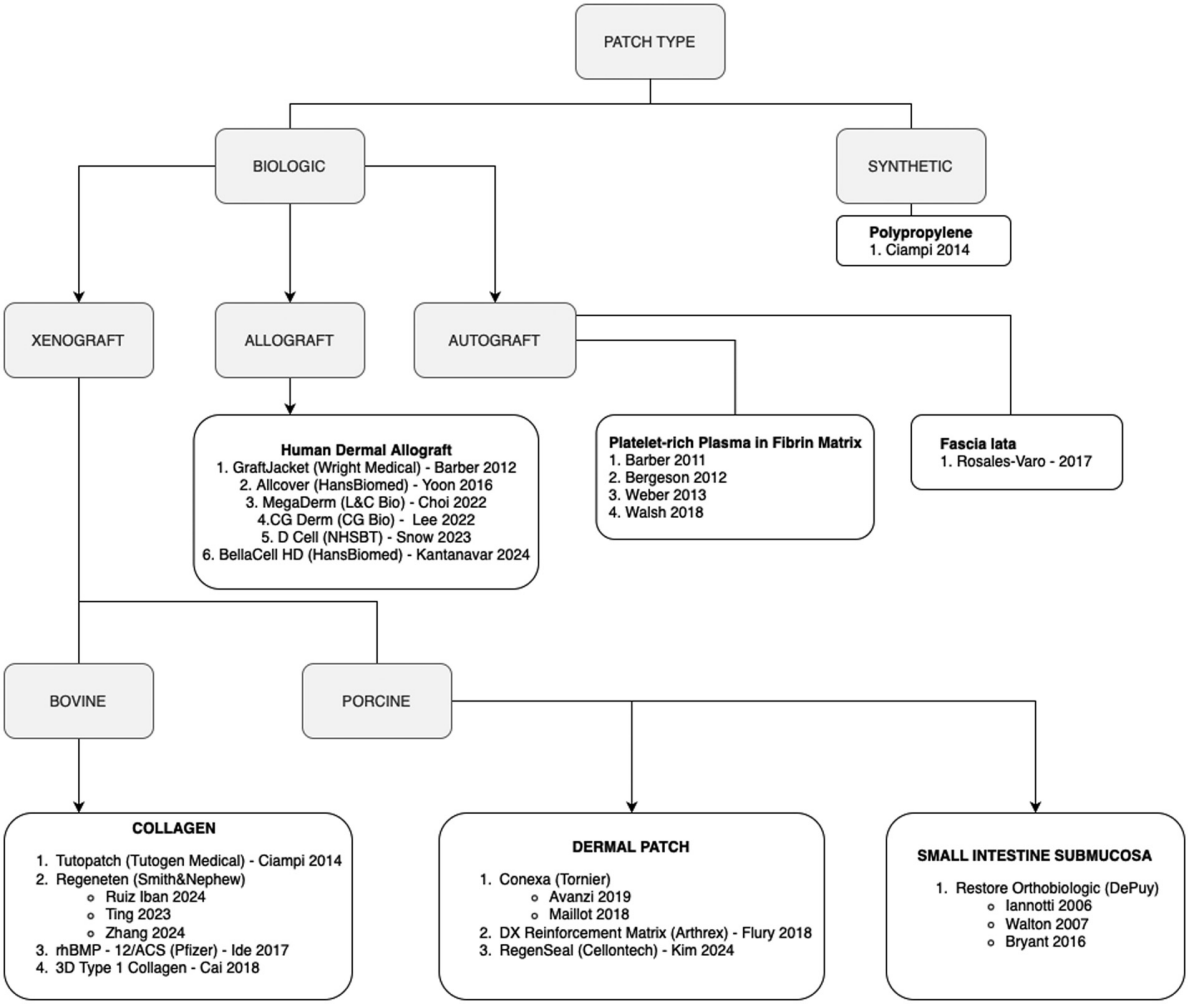
Breakdown of augment type classification.

Of the 24 studies, 12 were RCTs^††^ and 12 were cohort studies^‡‡^ (Table 1). A total of 1470 patients (1471 shoulders) were included across all studies, with individual sample sizes ranging from 20 to 167 patients. Almost all studies (n = 20) included only patients undergoing primary RCR.^§§^ One study consisted only of revision cases,^76^ 1 study analyzed primary and revision cases,^84^ and 2 studies did not report if the repair was primary or revision.^20,79^ All studies evaluated structural augments as the intervention. For the purposes of this review, augments were classified by type (Figure 2). All studies evaluated biologic augments. One study evaluated a biologic augment and a synthetic augment (2 separate intervention groups).^20^ Biologic augments were classified as xenograft, allograft, or autograft: 13 studies evaluated xenografts,^‖‖^ 6 allografts,^5,17,37,46,71,82^ and 5 autografts.^6,8,63,78,81^ Of the 13 studies that evaluated xenografts, 6 examined bovine collagen,^12,20,35,64,76,84^ 4 porcine dermis,^2,30,40,51^ and 3 porcine small intestine submucosa.^11,34,79^ All studies utilized standard RCR as the comparator.

**Table 1:** Study Characteristics*^*a*^*

Author (Year)	Study Design	LoE	Sample Size	Primary or Revision	Intervention	Comparator
Avanzi (2019)^2^	RCT	2	92 (I: 46, C: 46)	Primary	Porcine dermal patch (Conexa; Tornier)	Single-row RCR
Barber (2011)^6^	PCS	3	40 (I: 20, C: 20)	Primary	Cascade PRP fibrin matrix construct (Musculoskeletal Transplant Foundation)	Single-row RCR
Barber (2012)^5^	RCT	2	42 (I: 22, C: 20)	Primary	Human dermal allograft (GraftJacket; Wright Medical Technology)	RCR
Bergeson (2012)^8^	PCS	3	37 (I: 16, C: 21)	Primary	PRP in fibrin matrix	Single- and double-row RCR
Bryant (2016)^11^	RCT	2	62 (I: 34, C: 28)	Primary	Porcine small intestine submucosa implant (Restore Orthobiologic Implant; DePuy Orthopaedics)	RCR
Cai (2018)^12^	RCT	2	112 (I: 54, C: 58)	Primary	3-dimensional type I bovine collagen (Zhejiang Xingyue Biotechnology)	Double-row RCR
Choi (2022)^17^	Matched RCS	3	34 (I: 17, C: 17)	Primary	Human dermal allograft (MegaDerm; L&C Bio)	RCR
Ciampi (2014)^20^	RCS	3	152 (I: 101,*^*b*^* C: 51)	NR	Synthetic group: nonabsorbable polypropylene patch (Repol Angimesh; Angiologica). Biological group: absorbable bovine collagen patch (TUTOPATCH; Tutogen Medical GmbH)	Single-row RCR
Flury (2018)^30^	Matched PCS	3	40 (I: 20, C: 20)	Primary	Porcine dermal extracellular matrix (DX Reinforcement Matrix; Arthrex)	Double-row RCR
Iannotti (2006)^34^	RCT	2	30 (I: 15, C: 15)	Primary	Porcine small intestine submucosa implant (Restore Orthobiologic Implant; DePuy Orthopaedics)	RCR
Ide (2017)^35^	RCT	2	20 (I: 16, C: 4)	Primary	Absorbable collagen sponge reinforced with rhBMP-12	RCR
Kantanavar (2024)^37^	RCS	3	167 (I: 36, C: 131)	Primary	Human dermal allograft (BellaCell HD; HansBiomed)	Single-row RCR
Kim (2024)^40^	RCT	2	55 (I: 29, C: 26)	Primary	Porcine dermal patch (RegenSeal; Cellontech)	Double-row RCR
Lee (2022)^46^	RCT	2	43 (I: 22, C: 21)	Primary	Human dermal allograft (CGDerm; CGBio)	Double-row RCR
Maillot (2018)^51^	PCS	3	23 (I: 11, C: 12)	Primary	Porcine dermal patch (Conexa; Tornier)	Single-row RCR
Rosales-Varo (2018)^63^	PCS	3	20 (I: 10, C:10)	Primary	Autologous fascia lata	Single-row RCR
Ruiz Ibán (2024)^64^	RCT	2	124 (I: 61, C: 63)	Primary	Bioinductive bovine collagen patch (REGENETEN; Smith & Nephew)	Double-row RCR
Snow (2023)^71^	RCT	2	40 (I: 20, C: 20)	Primary	Human dermal allograft	Double-row RCR
Ting (2023)^76^	Matched RCS	3	51 (I: 19, C: 32)	Revision	Bioinductive bovine collagen patch (REGENETEN; Smith & Nephew)	Single-row RCR
Walsh (2018)^78^	RCT	2	72 (I: 28, C: 44)	Primary	PRP in fibrin matrix	Double-row RCR
Walton (2007)^79^	Matched RCS	3	31 (I: 15,*^*c*^* C: 16)	NR	Porcine small intestine submucosa implant (Restore Orthobiologic Implant; DePuy Orthopaedics)	Conventional RCR
Weber (2013)^81^	RCT	2	60 (I: 30, C: 30)	Primary	PRP in fibrin matrix	Single-row RCR
Yoon (2016)^82^	RCS	3	75 (I: 21, C: 54)	Primary	Combined bone marrow stimulation and human dermal allograft (Allocover; HansBiomed)	RCR
Zhang (2024)^84^	Matched RCS	3	48 (I: 24, C: 24)	Primary (n = 31), revision (n = 17)	Bioinductive bovine collagen patch (REGENETEN; Smith & Nephew)	Double-row RCR (n = 19 per group), single-row RCR (n = 5 per group)

a C, control; I, intervention; LoE, level of evidence; NR, not reported; PCS, prospective cohort study; PRP, platelet-rich plasma; RCR, rotator cuff repair; RCS, retrospective cohort study; RCT, randomized controlled trial; rhBMP-12, recombinant human bone morphogenetic protein 12.

b Collagen, n = 49; polypropylene, n = 52.

c Shoulders, n = 16.

The weighted mean age across studies was 61.7 years for the intervention and control groups (ranges, 55.3-68.3 and 55.3-67.1 years, respectively), with 40.9% (range, 10.5%-70%) and 45.8% (range, 20%-70%) of patients being female in the intervention and control groups (Table 2). Tear sizes ranged from small to massive.

**Table 2:** Patient Demographics*^*a*^*

	Age, y, Mean ± SD (Range)		Sex: Female, %		Tear Size, cm, Mean ± SD (Range)	
Author (Year)	Intervention	Control	Intervention	Control	Intervention	Control
Avanzi (2019)^2^	68*^*b*^* (58-78)	66*^*b*^* (54-76)	69.5	52.2	NR	NR
Barber (2011)^6^	57.6 (44-69)	57.8 (44-69)	30	35	14 medium (<3 cm) 6 large (≥3 cm)	14 medium (<3 cm) 6 large (≥3 cm)
Barber (2012)^5^	56 (43-69)	56 (34-72)	18	35	AP: 3.1 ± 1.4 ML: 3.2 ± 1.3	AP: 2.4 ± 1.0 ML: 2.5 ± 1.1
Bergeson (2012)^8^	65 ± 7	65 ± 9	NR	NR	3.8 ± 1.1*^*c*^*	3.9 ± 1.1*^*c*^*
Bryant (2016)^11^	55.3 ± 11.3	58.3 ± 10.2	14.7	78.6	AP: 3.1 ± 1.4 ML: 2.6 ± 1.1	AP: 2.9 ± 1.2 ML: 2.9 ± 1.1
Cai (2018)^12^	62.9 ± 9.7	61.3 ± 7.6	53	35	4.6 ± 1.6*^*c*^*	4.4 ± 1.1*^*c*^*
Choi (2022)^17^	68.3 ± 6.3	55.3 ± 11.3	41	41	AP: 2.9 ± 0.6 ML: 3.2 ± 0.7	AP: 3.0 ± 0.6 ML: 3.3 ± 0.8
Ciampi (2014)^20^	C: 66.5 (58-76) P: 66.2 (57-77)	67.1 (58-77)	C: 22 P: 21	31	NR	NR
Flury (2018)^30^	67.6 ± 3.1	65.4 ± 3.3	70	70	NR	NR
Iannotti (2006)^34^	58	57	27	20	4 large (4-5cm) 11 massive (>5cm)	5 large (4-5cm) 10 massive (>5cm)
Ide (2017)^35^	60.6 ± 5.7	62.3 ± 4.1	37.5	50	2.6 ± 0.5*^*c*^*	3.1 ± 0.6*^*c*^*
Kantanavar (2024)^37^	64 (49-80)	66 (41-81)	58.3	55.7	23 large (3-5cm) 13 massive (>5cm)	93 large (3-5cm) 38 massive (>5cm)
Kim (2024)^40^	62.5 ± 7.0	61.9 ± 8.2	41.4	34.6	11 medium (<3 cm) 18 large (3-5 cm)	10 medium (<3 cm) 16 large (3-5 cm)
Lee (2022)^46^	60.2 ± 8.4	58.3 ± 7.0	68.2	66.7	AP: 3.0 ± 0.5 ML: 3.5 ± 0.6	AP: 3.1 ± 0.5 ML: 3.3 ± 0.4
Maillot (2018)^51^	56*^*b*^* (46-63)	58*^*b*^* (45-71)	54.5	58.3	AP: 3.7 ± 0.9 ML: 4.4 ± 0.8	AP: 3.5 ± 0.8 ML: 3.9 ± 0.6
Rosales-Varo (2018)^63^	57.0 ± 4.3	60.1 ± 3.1	60.0	60.0	Tear size area: 1.8 ± 0.6 cm^2^	Tear size area: 2.2 ± 0.7 cm^2^
Ruiz Ibán (2024)^64^	56.6 ± 6.86	58.7 ± 8.39	49.2	52.4	AP: 2.0 ± 0.7 ML: 1.7 ± 0.9	AP: 2.0 ± 0.6 ML: 1.4 ± 0.7
Snow (2023)^71^	62.4 (47-73)	62 (40-79)	40.0	40.0	3.0 ± 1.0*^*c*^*	2.4 ± 0.9*^*c*^*
Ting (2023)^76^	56 (38-69)	56 (39-68)	10.5	37.5	Tear size area: 2.1 (0.6-6.0) cm^2^	Tear size area: 2.7 (0.7-6.0) cm^2^
Walsh (2018)^78^	56.9 ± 6.7	54.9 ± 9.3	42.9	43.2	AP: 2.3 ± 0.7 ML: 1.9 ± 0.5	AP: 2.3 ± 0.8 ML: 2.1 ± 0.6
Walton (2007)^79^	60.2 ± 3.5	59.6 ± 3.1	33.3	31.3	Tear size area: 9.1 ± 1.6 cm^2^	Tear size area: 8.9 ± 1.9 cm^2^
Weber (2013)^81^	59.7 ± 8.2	64.5 ± 8.6	33.3	53.3	1.8 ± 0.8*^*c*^*	1.7 ± 1.2*^*c*^*
Yoon (2016)^82^	65.0 ± 8.7	62.8 ± 6.7	57.1	51.9	AP: 4.81 ± 0.54 cm ML: 3.61 ± 0.54 cm	AP: 4.70 ± 0.57 cm ML: 3.35 ± 0.49 cm
Zhang (2024)^84^	60 (40-73)	61 (47-77)	29.2	20.8	Tear size area: 15 cm^2^	Tear size area: 11 cm^2^

a AP, anteroposterior; C, collagen; ML, mediolateral; NR, not reported; P, polypropylene.

b Median.

c AP or ML not specified.

### Quality Assessment

Complete quality assessments can be found Appendix 2 (available online). According to the Cochrane RoB-2 tool for RCTs, only 2 of the 12 studies were judged to be at low risk of bias^40,64^ (Appendix 2, Figure A1). Three were at high risk of bias,^12,46,81^ and 7 had some concerns for risk of bias.^2,5,11,34,35,71,78^ Domains 1, 2, and 5 were most often rated down for risk of bias owing to a lack of adequate reporting of randomization methods (5 studies), lack of participant blinding (4 studies), and lack of prospective registration on trial registries (8 studies), respectively.

Based on the MINORS score for observational studies, 4 of 12 studies were judged as good quality^6,8,30,37^ and 8 as moderate quality^17,20,51,63,76,79,82,84^ (Appendix 2, Table A1, available online). Most points lost were due to a lack of unbiased assessment of the study endpoint (9 studies), prospective calculation of the study size (10 studies), and adequate statistical analyses (10 studies).

### Primary Outcome: Retears

**Figure 3.**
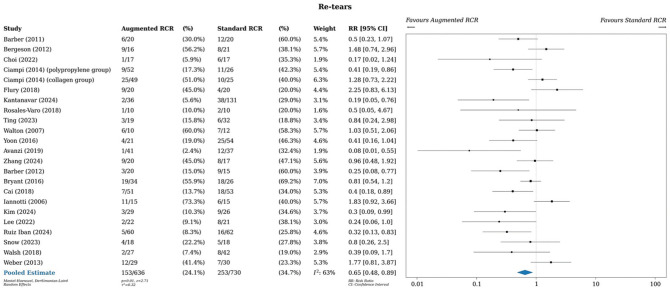
Meta-analysis of retears. RCR, rotator cuff repair; RR, risk ratio.

A meta-analysis of all studies that reported retear rates (22 studies, 23 comparisons) showed a risk ratio of 0.65 (95% CI, 0.48-0.89), displaying a statistically significant 35% decrease in the risk of retear in the intervention group (*P* = .01) (Figure 3).^¶¶^ Retears were confirmed radiographically by magnetic resonance imaging or ultrasound. Statistical heterogeneity was moderate, with an *I*^2^ value of 63%.

A sensitivity analysis omitting studies that used the Restore Orthobiologic Implant^11,34,79^ was performed, as this product has since been withdrawn from the market for severe autoimmune responses and poor outcomes. This analysis showed a risk ratio of 0.57 (95% CI, 0.39-0.82), displaying an even greater decrease of 43% in the risk of retear in the intervention group (Supplementary Figure S1). Similarly, a sensitivity analysis including only studies evaluating primary RCR showed a risk ratio of 0.57 (95% CI, 0.38-0.85), also displaying a greater decrease of 43% in the risk of retear in the intervention group (Supplementary Figure S2).

Subgroup analysis by study design demonstrated an overall risk ratio of 0.75 (95% CI, 0.51-1.12) for observational studies and 0.54 (95% CI, 0.32-0.9) for RCTs (Supplementary Figure S3). In both cases, the pooled estimate favors augmented RCR (25% and 46% risk decrease, respectively); however, the upper bound of the 95% CI for the observational studies crosses the line of no effect, suggesting uncertainty of the true effect.

Subgroup analysis by augment type demonstrated that the allograft group showed the greatest treatment benefit favoring augmented RCR, with a pooled estimate of 0.34 (95% CI, 0.21-0.56), displaying a 66% decrease in the risk of retear in the intervention group (Supplementary Figure S4). Subgroup analysis of the xenograft augments by bovine and porcine showed no difference in risk ratio for both when compared with standard RCR (Supplementary Figure S5).

### Pain Score

**Figure 4.**
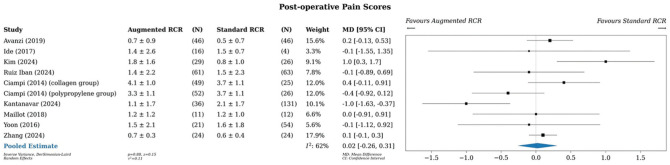
Meta-analysis of pain scores. MD, mean difference; RCR, rotator cuff repair.

A meta-analysis of all studies that reported pain scores (9 studies, 10 comparisons) showed an MD of 0.02 points (95% CI, −0.26 to 0.31) in favor of standard RCR, although this finding was not significant (Figure 4).^##^ Statistical heterogeneity was moderate, with an *I*^2^ value of 62%.

Subgroup analysis by study design demonstrated no difference (Supplementary Figure S6). Subgroup analysis by augment type revealed that the xenograft group showed a slight benefit in favor of standard RCR (MD, 0.21; 95% CI, 0.01-0.41), although this difference is trivial (Supplementary Figure S7).

### American Shoulder and Elbow Surgeons Score

**Figure 5.**
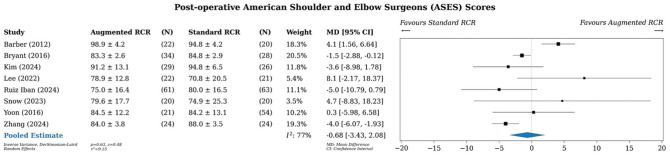
Meta-analysis of ASES scores. ASES, American Shoulder and Elbow Surgeons; MD, mean difference; RCR, rotator cuff repair.

A meta-analysis of all studies that reported American Shoulder and Elbow Surgeons (ASES) scores (n = 8) showed an MD of −0.68 points (95% CI, –3.43 to 2.08) in favor of standard RCR, although this finding was not significant (Figure 5).^5,11,40,46,64,71,82,84^ Statistical heterogeneity was moderate, with an *I*^2^ value of 77%.

Subgroup analysis by study design demonstrated no difference (Supplementary Figure S8). Subgroup analysis by augment type revealed that the xenograft group showed a slight benefit in favor of standard RCR (MD, −1.63; 95% CI, −2.97 to −0.29), although this difference is trivial (Supplementary Figure S9).

### Constant Score

**Figure 6.**
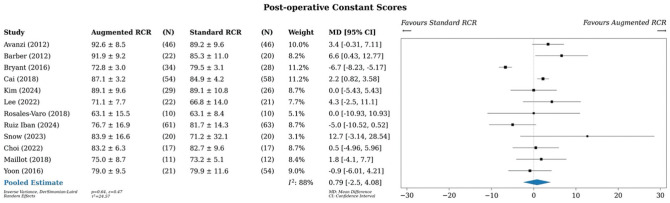
Meta-analysis of Constant scores. MD, mean difference; RCR, rotator cuff repair.

A meta-analysis of all studies that reported Constant scores (n = 12) showed an MD of 0.79 points (95% CI, –2.5 to 4.08) in favor of augmented RCR, although this finding was not significant (Figure 6).^***^ Statistical heterogeneity was high, with an *I*^2^ value of 88%.

Subgroup analyses demonstrated no differences across study design or augment type (Supplementary Figures S10 and S11).

## Discussion

### Key Findings

The results of this systematic review suggest that in patients undergoing RCR for full-thickness rotator cuff tears, the use of structural augmentation results in a 35% decrease in retear rate when compared with standard RCR. Subgroup analysis by study design demonstrated a greater reduction in retear rate in RCTs as compared with observational studies. Subgroup analysis by augment type revealed the lowest retear rates in allografts as compared with other graft types. No significant differences were seen between standard RCR and augmented RCR with regard to the short-term PROs, such as pain, ASES, and Constant scores.

### Relation to Other Work

There has been increased interest in the use of structural augmentation for RCR over the last decade. The findings of this review add to the growing body of evidence supporting the use of structural augmentation to improve structural healing after RCR. In a recent systematic review, Orozco et al^60^ analyzed 6 RCTs evaluating patch augmentation for RCR. Their findings align with those of the present review, displaying a reduction in retear rates in the augmentation group. However, they did not include subgroup analyses by graft type and had only 6 RCTs, while the present review examined 11.

In a systematic review of 7 studies, de Andrade et al^1^ found a significant reduction in retear rates in patients who received a patch augmentation. While the results of the present review showed a significant reduction in retear rate, the effect size (0.65; 95% CI, 0.48-0.89) represented a smaller reduction in retears than that reported by de Andrade et al (0.32; 95% CI, 0.18-0.55), with reductions of 35% and 68%, respectively. This difference may be due to the inclusion of a greater number of studies in the current review, allowing for more robust statistical analyses.

An important objective of this review was to compare retear rates among graft types. To date, this is the first review that has been able to perform a comparative meta-analysis stratified by graft type. The use of allograft appears to result in the lowest retear rate (0.34; 95% CI, 0.21-0.56). The low heterogeneity (*I*^2^ = 0%) observed among studies using allograft strengthens the confidence in these findings. Similar findings were observed in a review by Ferguson et al,^29^ which demonstrated intact repairs in 85% of patients who received allograft augments. Baldwin et al^4^ conducted a meta-analysis that mirrored our findings, showing a robust 66% reduction in retear rate in the allograft subgroup, which was superior to synthetic grafts in their review. Only 1 comparative study evaluating synthetic augments was available in the literature and included in this review.^20^ More comparative studies are needed to further evaluate the role of synthetic grafts for RCR augmentation.

Reviews by Mandalia et al^53^ and Steinhaus et al^72^ concluded that allografts showed the most promise in adding structural support to the repair construct. The positive influence of allografts on tendon repair may be 2-fold, owing to the mechanical strength provided by the augment in the early postoperative period and possibly to their biologic constituents (collagen, chondroitin sulphate, etc).^38^

Although strong support exists in the literature for allografts, evidence for augmentation of rotator cuff tears with xenografts is not compelling. A review by Ono et al^59^ found inferior healing rates of RCR augmented with xenografts as compared with other graft material, although their study included data primarily from retrospective case series. In a more recent review of comparative studies, there were no significant differences in failure rates between xenograft augmentation and standard RCR, and the xenograft group even showed a significantly higher complication rate.^57^ There is significant variability among xenograft patch designs, which include patches derived from porcine dermis, porcine small intestine submucosa, and bovine collagen, which makes it difficult to make strong conclusions about xenografts as a single subtype. Bovine collagen implants have shown improvements in clinical outcomes and reduction in retear rates in recent studies, although further research is required solidify these findings.^10,80^ Several early studies used xenograft small intestine submucosa patches, and because of the disappointing healing rates and a concerning incidence of inflammatory reactions, they have been largely abandoned for use in rotator cuff augmentation.^3,14,21,45^ Results published from individual studies using xenograft dermal grafts have been mixed.^14^ While some studies reported improvements in PRO scores and healing rates,^22,30^ a study by Maillot et al^51^ found no difference in outcomes and a higher complication rate in the patch group as compared with standard repair. It appears that dermal xenografts do not suffer from sterile inflammatory responses, but it is not clear that they provide much benefit either.^14^ The present review supports those findings, as there appeared to be no difference in retear rates between the use of xenograft augmentation and standard RCR.

### Implications for Clinical and Research

The current review aimed to determine whether there were any differences in PROs. While the review by de Andrade et al^1^ showed lower pain scores and significant improvement in the UCLA (University of California Los Angeles) shoulder rating scale in the patch augmentation group as compared with the control group, the present review found no differences in any PROs between the groups overall. There was substantial heterogeneity among the studies with regard to all analyzed PROs (*I*^2^ range, 62%-88%), and this was explored through subgroup analysis. While this heterogeneity was partially explained by graft type, no graft type demonstrated any difference between the augmented and standard RCR groups that remotely approached the minimal clinically important difference of any of the observed outcomes.^49,52,73^

The correlation between the structural integrity of RCR and PROs remains controversial.^65^ After RCR, the majority of any functional improvements are expected to have occurred by 1 year postoperatively, and it appears that patients with retears do similar clinically as compared with patients with intact repairs.^16,48,65^ Most of the studies in this review reported outcomes at 1 year postoperatively, and the findings appear to support that patients with retears can still achieve satisfactory functional outcomes. However, the primary goal of RCR is typically a healed interface between tendon and bone.^14^ Various studies have demonstrated that if an RCR heals, positive outcomes are usually sustained long-term.^13^ Some evidence suggests that rotator cuff retears can increase in size over longer periods and PROs can deteriorate, particularly in regard to strength.^26,65^ There has been considerable research focus and investment to develop strategies to increase the likelihood of tendon healing. Based on the current findings, it appears that structural augmentation may help achieve this goal. Yet, it is still unclear whether augmentation results in improved functional outcomes for patients, and studies with longer-term follow-up may be required to fully address this question.

### Strengths and Limitations

This review has several strengths, such as the comprehensive search strategy, sample size, thorough quality assessments, and rigorous methodology. Moreover, the ability to analyze level 1 RCTs in isolation is a unique strength of this study. There are also limitations to this systematic review and meta-analysis: the inclusion of trials with small sample sizes, between-study heterogeneity, variability in surgical techniques, and inconsistent reporting of tear characteristics. Many of the RCTs displayed effects with wide confidence intervals attributed to small sample sizes. By including 11 total RCTs, this review was able to provide a more precise estimate. Heterogeneity was present across most of the meta-analyses, including retear rate (*I*^2^ = 63%), pain scores (*I*^2^ = 62%), ASES scores (*I*^2^ = 77%), and Constant scores (*I*^2^ = 88%). This was explored through subgroup analyses defined a priori and explained in part by study design and graft type. There was variability in the length of final follow-up reported, with 5 studies not reporting a specific time point. However, the majority of the studies (15/24) reported a minimum follow-up of at least 1 year. With regard to the augments, there were various proprietary patches within the defined subgroups of autograft, allograft, and xenograft (Figure 2). Because of inconsistent reporting, this review was unfortunately unable to provide clarity on the effect of structural augmentation depending on specific nuances in surgical technique or tear size.

## Conclusion

In summary, the findings of the present systematic review and meta-analysis suggest a significant reduction in retear rate with the use of structural augmentation in RCR. However, it does not appear that structural augmentation results in any improvements in PROs in comparison with standard RCR. When graft types were analyzed separately, the use of xenograft did not appear to improve any clinical outcomes, while allografts resulted in the lowest rate of retear. Further research is needed to clarify which patient factors and tear characteristics may influence the effectiveness of structural patch augmentation.

## Supplemental Material

sj-docx-1-ajs-10.1177_03635465251400356 – Supplemental material for Structural Augmentation in Rotator Cuff Repair Decreases the Risk of Retear: A Systematic Review and Meta-analysis Supplemental material, sj-docx-1-ajs-10.1177_03635465251400356 for Structural Augmentation in Rotator Cuff Repair Decreases the Risk of Retear: A Systematic Review and Meta-analysis by Danielle Dagher, Darshil Shah, Colin Kruse, Hassaan Abdel Khalik, Usama Saleh, Abdul-Ilah Hachem, John M. Tokish, Asheesh Bedi and Moin Khan in The American Journal of Sports Medicine

sj-docx-10-ajs-10.1177_03635465251400356 – Supplemental material for Structural Augmentation in Rotator Cuff Repair Decreases the Risk of Retear: A Systematic Review and Meta-analysis Supplemental material, sj-docx-10-ajs-10.1177_03635465251400356 for Structural Augmentation in Rotator Cuff Repair Decreases the Risk of Retear: A Systematic Review and Meta-analysis by Danielle Dagher, Darshil Shah, Colin Kruse, Hassaan Abdel Khalik, Usama Saleh, Abdul-Ilah Hachem, John M. Tokish, Asheesh Bedi and Moin Khan in The American Journal of Sports Medicine

sj-docx-11-ajs-10.1177_03635465251400356 – Supplemental material for Structural Augmentation in Rotator Cuff Repair Decreases the Risk of Retear: A Systematic Review and Meta-analysis Supplemental material, sj-docx-11-ajs-10.1177_03635465251400356 for Structural Augmentation in Rotator Cuff Repair Decreases the Risk of Retear: A Systematic Review and Meta-analysis by Danielle Dagher, Darshil Shah, Colin Kruse, Hassaan Abdel Khalik, Usama Saleh, Abdul-Ilah Hachem, John M. Tokish, Asheesh Bedi and Moin Khan in The American Journal of Sports Medicine

sj-docx-12-ajs-10.1177_03635465251400356 – Supplemental material for Structural Augmentation in Rotator Cuff Repair Decreases the Risk of Retear: A Systematic Review and Meta-analysis Supplemental material, sj-docx-12-ajs-10.1177_03635465251400356 for Structural Augmentation in Rotator Cuff Repair Decreases the Risk of Retear: A Systematic Review and Meta-analysis by Danielle Dagher, Darshil Shah, Colin Kruse, Hassaan Abdel Khalik, Usama Saleh, Abdul-Ilah Hachem, John M. Tokish, Asheesh Bedi and Moin Khan in The American Journal of Sports Medicine

sj-docx-13-ajs-10.1177_03635465251400356 – Supplemental material for Structural Augmentation in Rotator Cuff Repair Decreases the Risk of Retear: A Systematic Review and Meta-analysis Supplemental material, sj-docx-13-ajs-10.1177_03635465251400356 for Structural Augmentation in Rotator Cuff Repair Decreases the Risk of Retear: A Systematic Review and Meta-analysis by Danielle Dagher, Darshil Shah, Colin Kruse, Hassaan Abdel Khalik, Usama Saleh, Abdul-Ilah Hachem, John M. Tokish, Asheesh Bedi and Moin Khan in The American Journal of Sports Medicine

sj-docx-2-ajs-10.1177_03635465251400356 – Supplemental material for Structural Augmentation in Rotator Cuff Repair Decreases the Risk of Retear: A Systematic Review and Meta-analysis Supplemental material, sj-docx-2-ajs-10.1177_03635465251400356 for Structural Augmentation in Rotator Cuff Repair Decreases the Risk of Retear: A Systematic Review and Meta-analysis by Danielle Dagher, Darshil Shah, Colin Kruse, Hassaan Abdel Khalik, Usama Saleh, Abdul-Ilah Hachem, John M. Tokish, Asheesh Bedi and Moin Khan in The American Journal of Sports Medicine

sj-docx-3-ajs-10.1177_03635465251400356 – Supplemental material for Structural Augmentation in Rotator Cuff Repair Decreases the Risk of Retear: A Systematic Review and Meta-analysis Supplemental material, sj-docx-3-ajs-10.1177_03635465251400356 for Structural Augmentation in Rotator Cuff Repair Decreases the Risk of Retear: A Systematic Review and Meta-analysis by Danielle Dagher, Darshil Shah, Colin Kruse, Hassaan Abdel Khalik, Usama Saleh, Abdul-Ilah Hachem, John M. Tokish, Asheesh Bedi and Moin Khan in The American Journal of Sports Medicine

sj-docx-4-ajs-10.1177_03635465251400356 – Supplemental material for Structural Augmentation in Rotator Cuff Repair Decreases the Risk of Retear: A Systematic Review and Meta-analysis Supplemental material, sj-docx-4-ajs-10.1177_03635465251400356 for Structural Augmentation in Rotator Cuff Repair Decreases the Risk of Retear: A Systematic Review and Meta-analysis by Danielle Dagher, Darshil Shah, Colin Kruse, Hassaan Abdel Khalik, Usama Saleh, Abdul-Ilah Hachem, John M. Tokish, Asheesh Bedi and Moin Khan in The American Journal of Sports Medicine

sj-docx-5-ajs-10.1177_03635465251400356 – Supplemental material for Structural Augmentation in Rotator Cuff Repair Decreases the Risk of Retear: A Systematic Review and Meta-analysis Supplemental material, sj-docx-5-ajs-10.1177_03635465251400356 for Structural Augmentation in Rotator Cuff Repair Decreases the Risk of Retear: A Systematic Review and Meta-analysis by Danielle Dagher, Darshil Shah, Colin Kruse, Hassaan Abdel Khalik, Usama Saleh, Abdul-Ilah Hachem, John M. Tokish, Asheesh Bedi and Moin Khan in The American Journal of Sports Medicine

sj-docx-6-ajs-10.1177_03635465251400356 – Supplemental material for Structural Augmentation in Rotator Cuff Repair Decreases the Risk of Retear: A Systematic Review and Meta-analysis Supplemental material, sj-docx-6-ajs-10.1177_03635465251400356 for Structural Augmentation in Rotator Cuff Repair Decreases the Risk of Retear: A Systematic Review and Meta-analysis by Danielle Dagher, Darshil Shah, Colin Kruse, Hassaan Abdel Khalik, Usama Saleh, Abdul-Ilah Hachem, John M. Tokish, Asheesh Bedi and Moin Khan in The American Journal of Sports Medicine

sj-docx-7-ajs-10.1177_03635465251400356 – Supplemental material for Structural Augmentation in Rotator Cuff Repair Decreases the Risk of Retear: A Systematic Review and Meta-analysis Supplemental material, sj-docx-7-ajs-10.1177_03635465251400356 for Structural Augmentation in Rotator Cuff Repair Decreases the Risk of Retear: A Systematic Review and Meta-analysis by Danielle Dagher, Darshil Shah, Colin Kruse, Hassaan Abdel Khalik, Usama Saleh, Abdul-Ilah Hachem, John M. Tokish, Asheesh Bedi and Moin Khan in The American Journal of Sports Medicine

sj-docx-8-ajs-10.1177_03635465251400356 – Supplemental material for Structural Augmentation in Rotator Cuff Repair Decreases the Risk of Retear: A Systematic Review and Meta-analysis Supplemental material, sj-docx-8-ajs-10.1177_03635465251400356 for Structural Augmentation in Rotator Cuff Repair Decreases the Risk of Retear: A Systematic Review and Meta-analysis by Danielle Dagher, Darshil Shah, Colin Kruse, Hassaan Abdel Khalik, Usama Saleh, Abdul-Ilah Hachem, John M. Tokish, Asheesh Bedi and Moin Khan in The American Journal of Sports Medicine

sj-docx-9-ajs-10.1177_03635465251400356 – Supplemental material for Structural Augmentation in Rotator Cuff Repair Decreases the Risk of Retear: A Systematic Review and Meta-analysis Supplemental material, sj-docx-9-ajs-10.1177_03635465251400356 for Structural Augmentation in Rotator Cuff Repair Decreases the Risk of Retear: A Systematic Review and Meta-analysis by Danielle Dagher, Darshil Shah, Colin Kruse, Hassaan Abdel Khalik, Usama Saleh, Abdul-Ilah Hachem, John M. Tokish, Asheesh Bedi and Moin Khan in The American Journal of Sports Medicine

## Notes

** References 2, 5, 6, 8, 11, 12, 17, 20, 30, 34, 35, 37, 40, 46, 51, 63, 64, 71, 76, 78, 79, 81, 82, 84

†† References 2, 5, 11, 12, 34, 35, 40, 46, 64, 71, 78, 81

‡‡ References 6, 8, 17, 20, 30, 37, 51, 63, 76, 79, 82, 84

§§ References 2, 5, 6, 8, 11, 12, 17, 30, 34, 35, 37, 40, 46, 51, 63, 64, 71, 78, 81, 82

‖‖ References 2, 11, 12, 20, 30, 34, 35, 40, 51, 64, 76, 79, 84

¶¶ References 2, 5, 6, 8, 11, 12, 17, 20, 30, 34, 37, 40, 46, 63, 64, 71, 76, 78, 79, 81, 82, 84

## References 2, 20, 35, 37, 40, 51, 64, 82, 84

*** References 2, 5, 11, 12, 17, 40, 46, 51, 63, 64, 71, 82

An online CME course associated with this article is available for 1 *AMA PRA* Category 1 Credit™ at https://education.sportsmed.org/Public/Catalog/Home.aspx?CourseSearch=1&Criteria=9&Option=25. In accordance with the standards of the Accreditation Council for Continuing Medical Education (ACCME), it is the policy of The American Orthopaedic Society for Sports Medicine that authors, editors, and planners disclose to the learners all financial relationships during the past 12 months with any commercial interest (A ‘commercial interest’ is any entity producing, marketing, re-selling, or distributing health care goods or services consumed by, or used on, patients). Any and all disclosures are provided in the online journal CME area which is provided to all participants before they actually take the CME activity. In accordance with AOSSM policy, authors, editors, and planners’ participation in this educational activity will be predicated upon timely submission and review of AOSSM disclosure. Noncompliance will result in an author/editor or planner to be stricken from participating in this CME activity.
