# Supplementary figures and images for: Structural Augmentation in Rotator Cuff Repair Decreases the Risk of Retear: A Systematic Review and Meta-analysis
Source: Am J Sports Med. 2026 Jan 18;54(6):1525–36. doi: 10.1177/03635465251400356 (PMC13133422; doi:10.1177/03635465251400356)

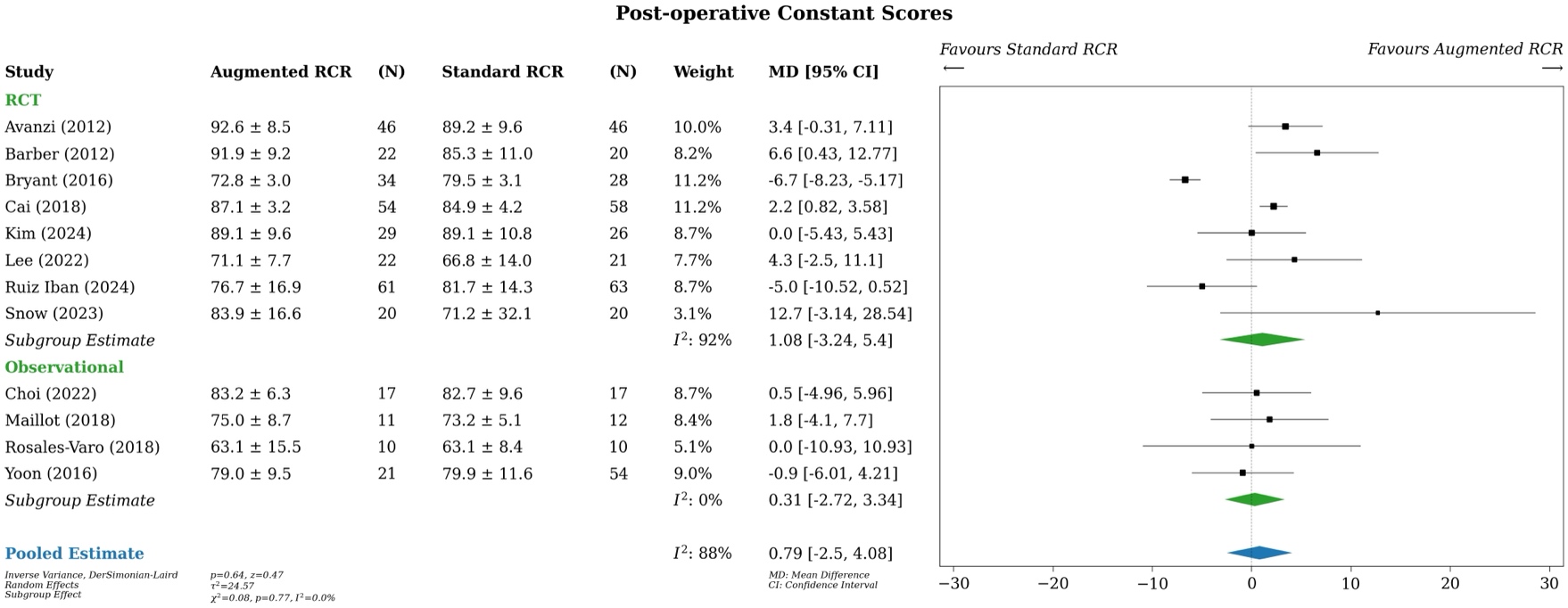


**Supplementary Figure 10.** Meta-analysis of Constant Scores (Study Design Subgroup)

Supplement: sj-docx-10-ajs-10.1177_03635465251400356 – Supplemental material for Structural Augmentation in Rotator Cuff Repair Decreases the Risk of Retear: A Systematic Review and Meta-analysis [file sj-docx-10-ajs-10.1177_03635465251400356.docx]

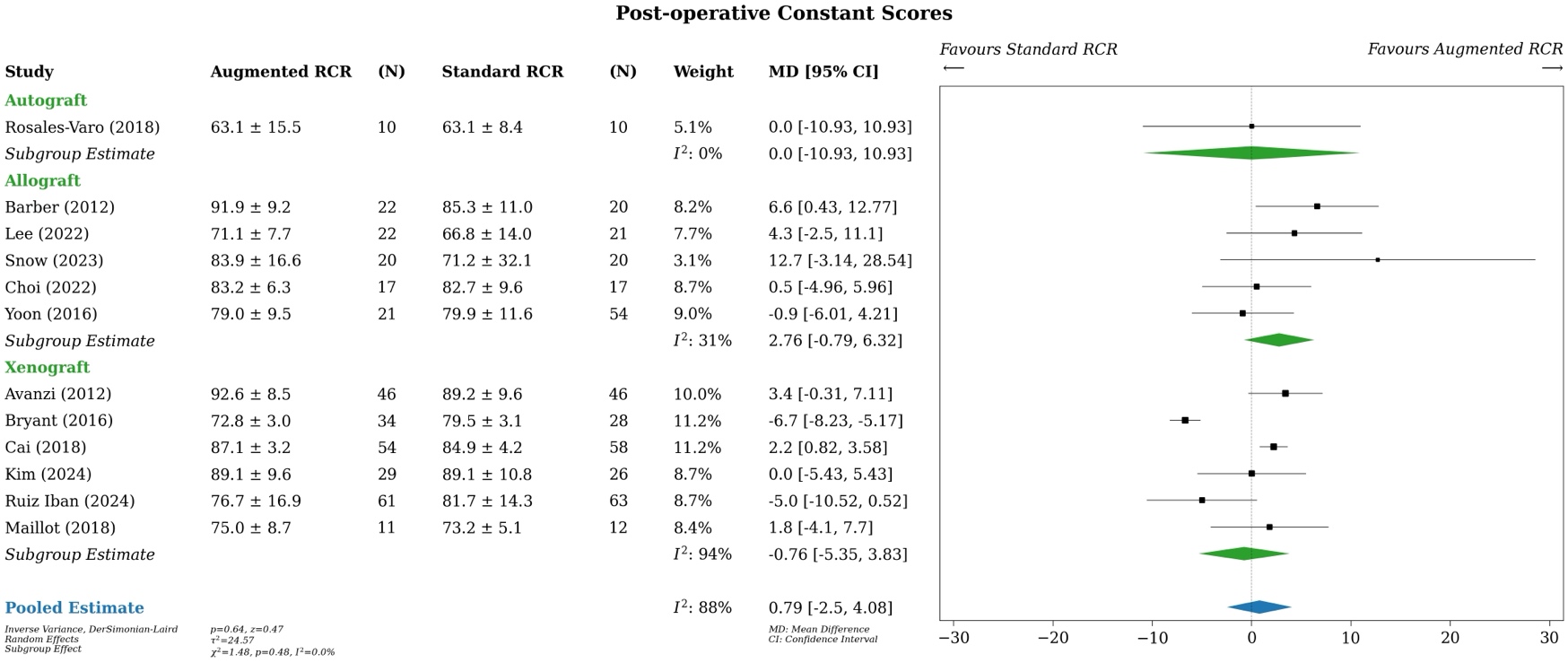


**Supplementary Figure 11.** Meta-analysis of Constant Scores (Augment Type Subgroup)

Supplement: sj-docx-11-ajs-10.1177_03635465251400356 – Supplemental material for Structural Augmentation in Rotator Cuff Repair Decreases the Risk of Retear: A Systematic Review and Meta-analysis [file sj-docx-11-ajs-10.1177_03635465251400356.docx]

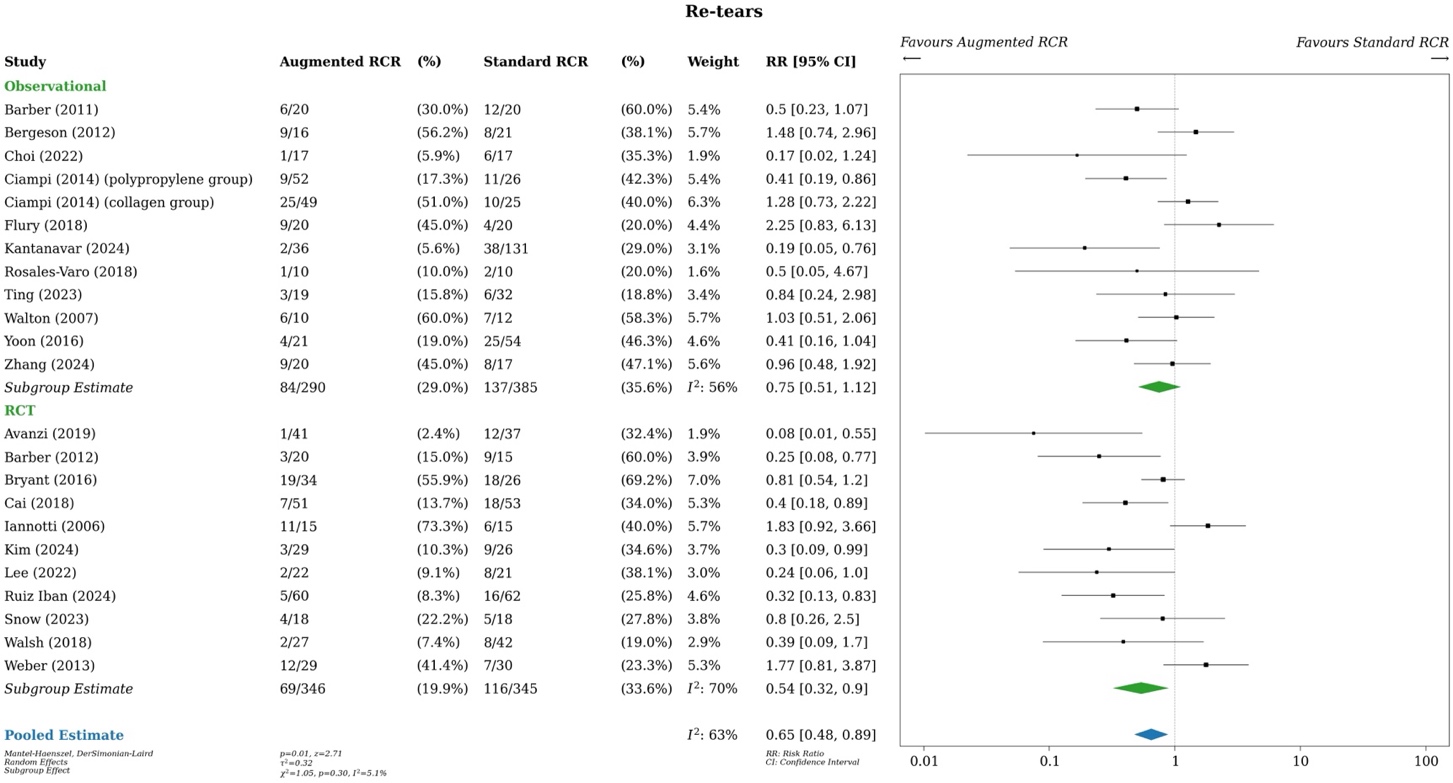


**Supplementary Figure 3.** Meta-analysis of Re-tears (Study Design Subgroup)

Supplement: sj-docx-3-ajs-10.1177_03635465251400356 – Supplemental material for Structural Augmentation in Rotator Cuff Repair Decreases the Risk of Retear: A Systematic Review and Meta-analysis [file sj-docx-3-ajs-10.1177_03635465251400356.docx]

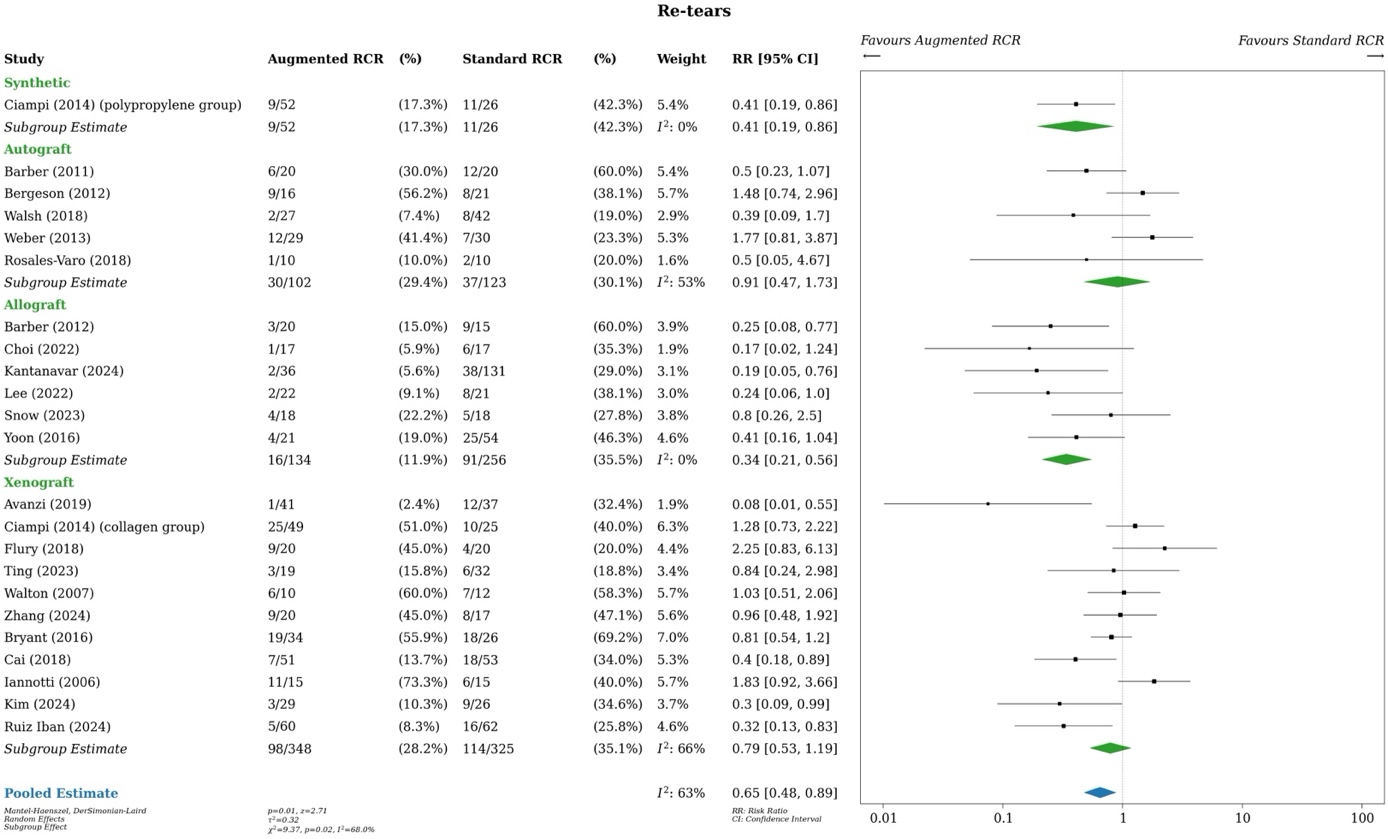


**Supplementary Figure 4.** Meta-analysis of Re-tears (Augment Type Subgroup)

Supplement: sj-docx-4-ajs-10.1177_03635465251400356 – Supplemental material for Structural Augmentation in Rotator Cuff Repair Decreases the Risk of Retear: A Systematic Review and Meta-analysis [file sj-docx-4-ajs-10.1177_03635465251400356.docx]

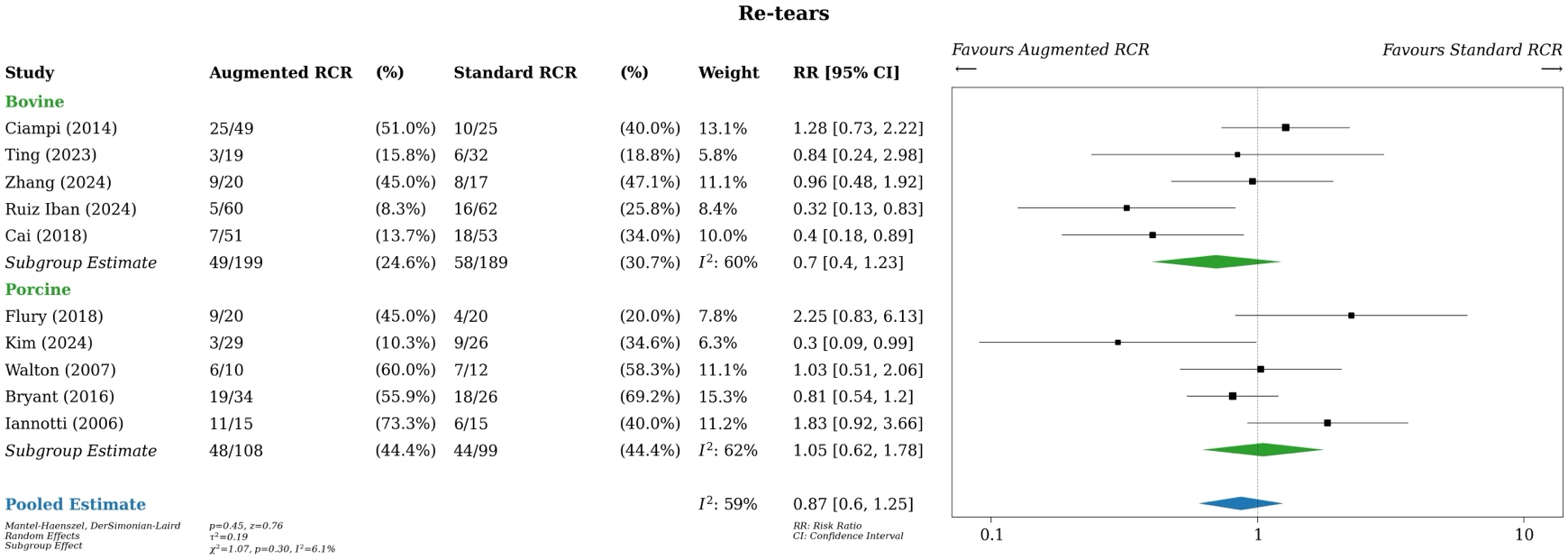


**Supplementary Figure 5.** Meta-analysis of Re-tears (Xenograft Type Subgroup)

Supplement: sj-docx-5-ajs-10.1177_03635465251400356 – Supplemental material for Structural Augmentation in Rotator Cuff Repair Decreases the Risk of Retear: A Systematic Review and Meta-analysis [file sj-docx-5-ajs-10.1177_03635465251400356.docx]

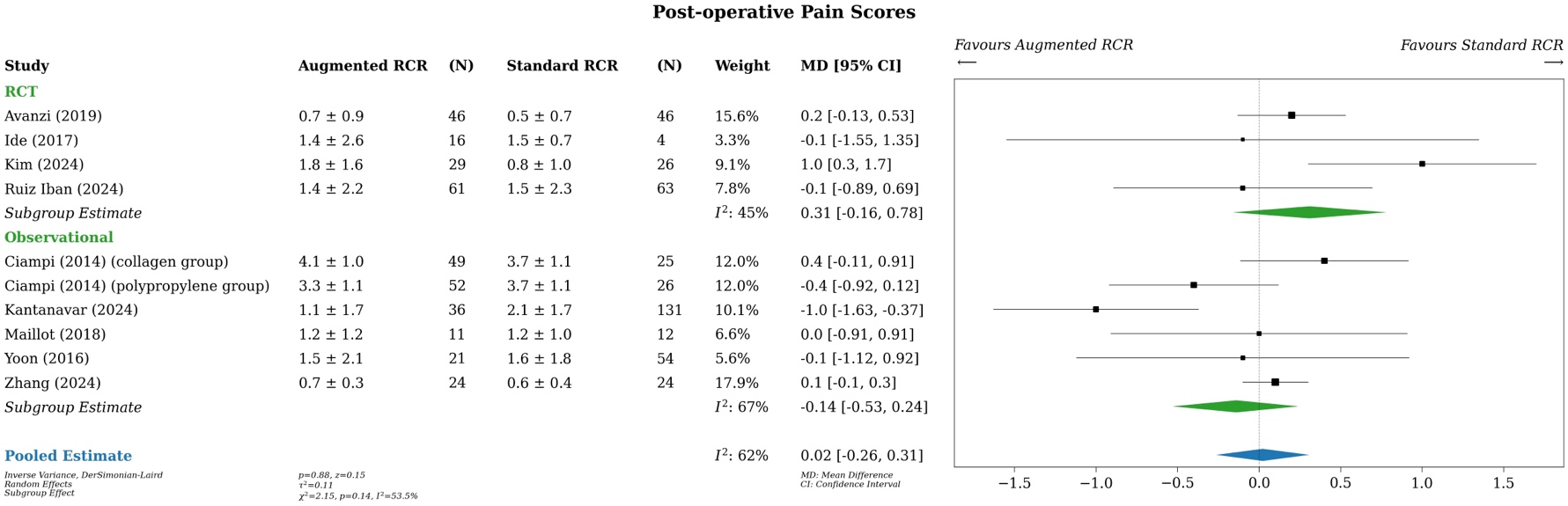


**Supplementary Figure 6.** Meta-analysis of Pain Scores (Study Design Subgroup)

Supplement: sj-docx-6-ajs-10.1177_03635465251400356 – Supplemental material for Structural Augmentation in Rotator Cuff Repair Decreases the Risk of Retear: A Systematic Review and Meta-analysis [file sj-docx-6-ajs-10.1177_03635465251400356.docx]

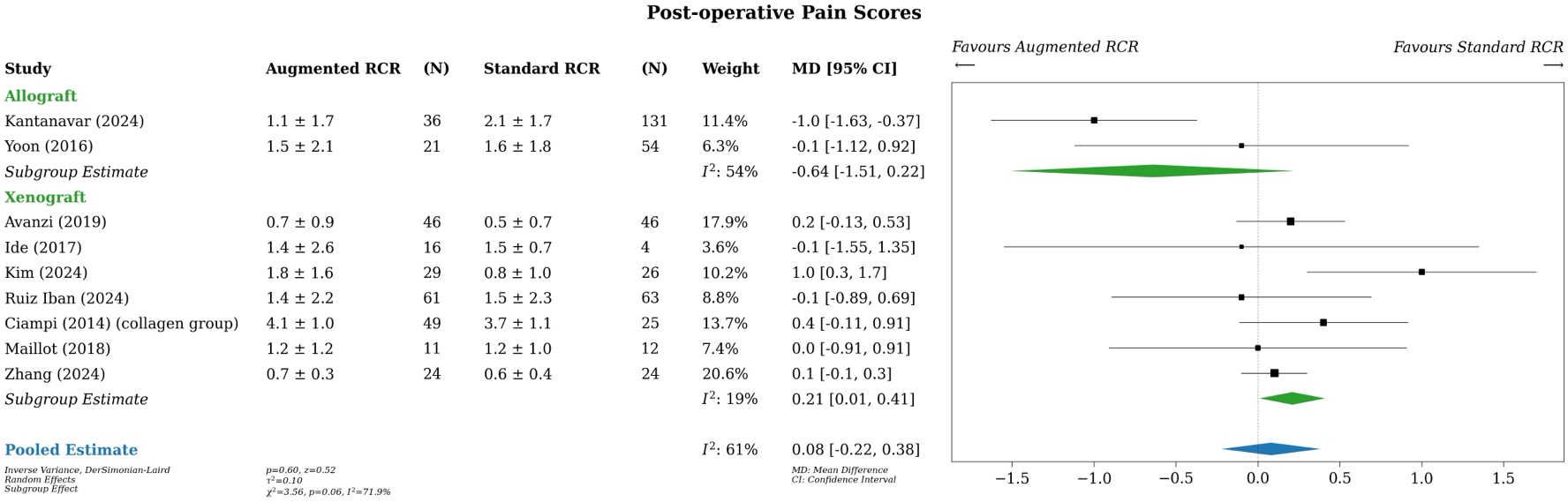


**Supplementary Figure 7.** Meta-analysis of Pain Scores (Augment Type Subgroup)

Supplement: sj-docx-7-ajs-10.1177_03635465251400356 – Supplemental material for Structural Augmentation in Rotator Cuff Repair Decreases the Risk of Retear: A Systematic Review and Meta-analysis [file sj-docx-7-ajs-10.1177_03635465251400356.docx]

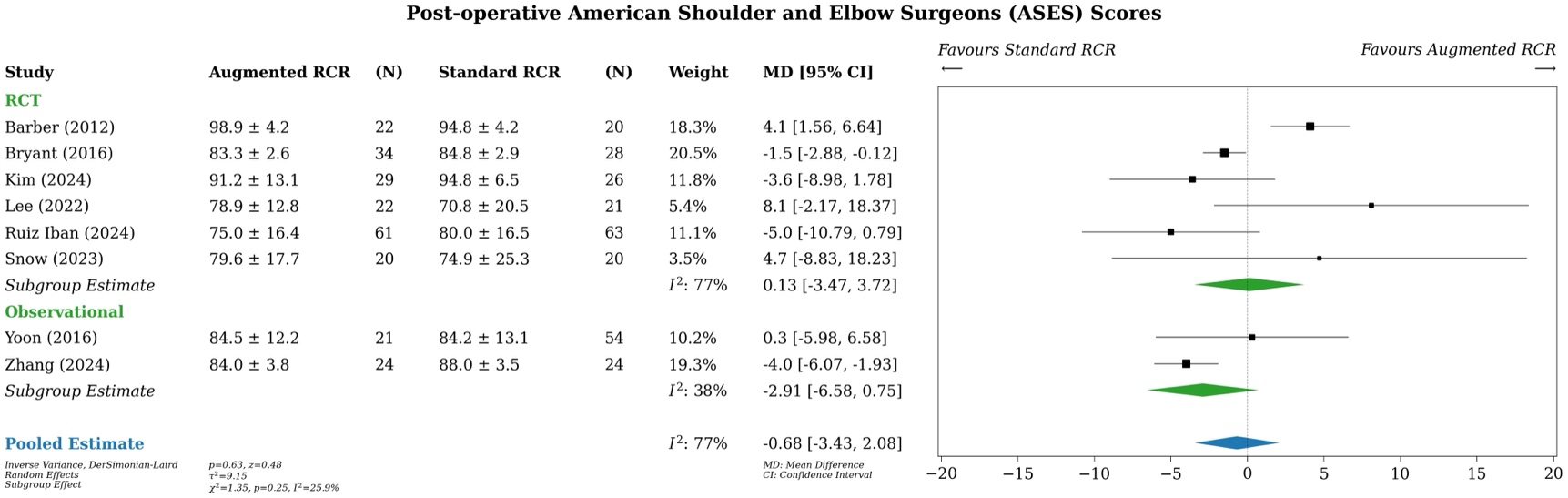


**Supplementary Figure 8.** Meta-analysis of ASES Scores (Study Design Subgroup)

Supplement: sj-docx-8-ajs-10.1177_03635465251400356 – Supplemental material for Structural Augmentation in Rotator Cuff Repair Decreases the Risk of Retear: A Systematic Review and Meta-analysis [file sj-docx-8-ajs-10.1177_03635465251400356.docx]

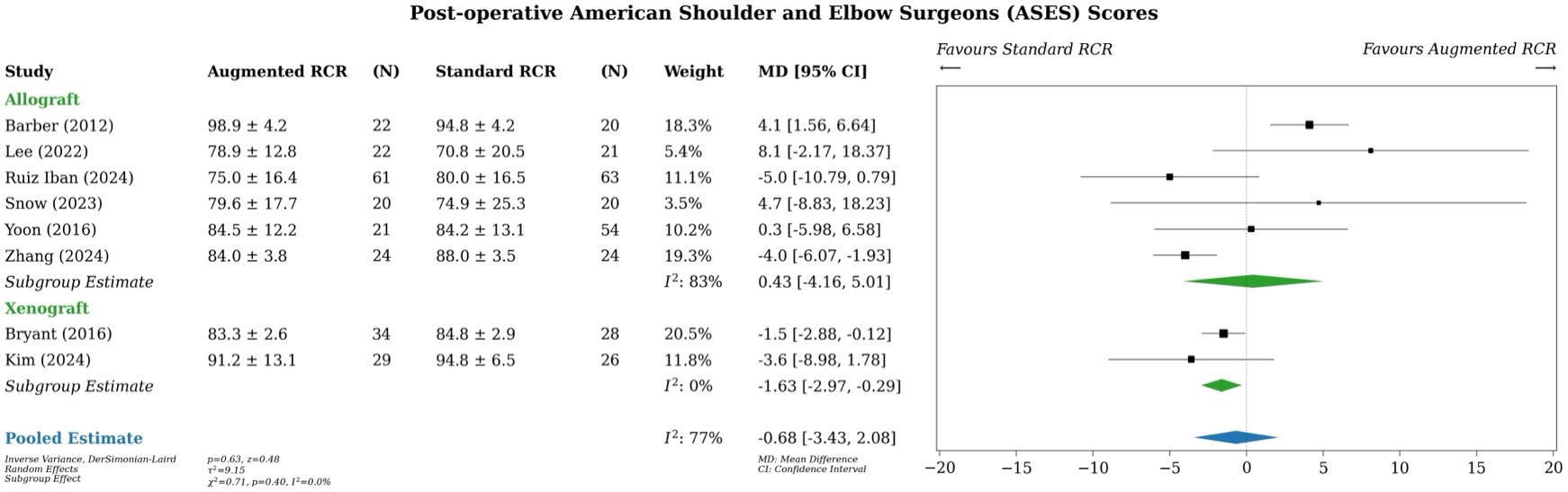


**Supplementary Figure 9.** Meta-analysis of ASES Scores (Augment Type Subgroup)

Supplement: sj-docx-9-ajs-10.1177_03635465251400356 – Supplemental material for Structural Augmentation in Rotator Cuff Repair Decreases the Risk of Retear: A Systematic Review and Meta-analysis [file sj-docx-9-ajs-10.1177_03635465251400356.docx]
