# Supplementary material for: Structural Augmentation in Rotator Cuff Repair Decreases the Risk of Retear: A Systematic Review and Meta-analysis
Source: Am J Sports Med. 2026 Jan 18;54(6):1525–36. doi: 10.1177/03635465251400356 (PMC13133422; doi:10.1177/03635465251400356)
Supplement: sj-docx-13-ajs-10.1177_03635465251400356 – Supplemental material for Structural Augmentation in Rotator Cuff Repair Decreases the Risk of Retear: A Systematic Review and Meta-analysis [file sj-docx-13-ajs-10.1177_03635465251400356.docx]

**Appendix B**. Quality Assessments


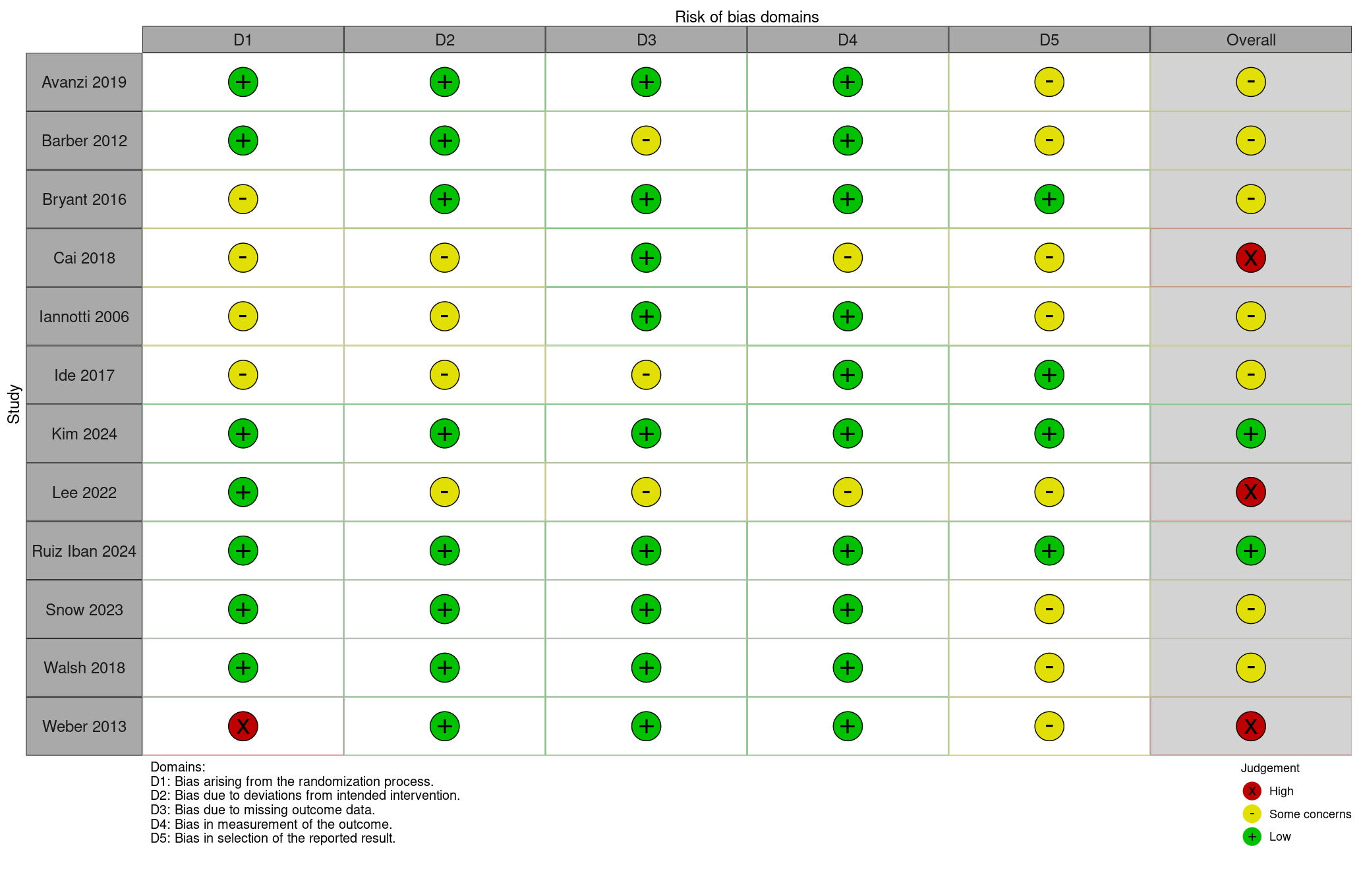


**Figure A1.** Risk of Bias Assessment of Included RCTs

**Table A1.** Methodological Index for Non-randomized Studies (MINORS) Scores for Observational Studies

| Author (year) | Item 1 | Item 2 | Item 3 | Item 4 | Item 5 | Item 6 | Item 7 | Item 8 | Item 9 | Item 10 | Item 11 | Item 12 | Overall score | Quality Judgment |
| --- | --- | --- | --- | --- | --- | --- | --- | --- | --- | --- | --- | --- | --- | --- |
| Barber (2011) | 2 | 2 | 1 | 2 | 2 | 1 | 2 | 2 | 2 | 2 | 2 | 1 | 21 | Good |
| Bergeson (2012) | 2 | 2 | 2 | 2 | 2 | 2 | 2 | 0 | 2 | 1 | 2 | 2 | 21 | Good |
| Choi (2022) | 2 | 2 | 2 | 2 | 0 | 2 | 2 | 0 | 2 | 2 | 2 | 1 | 19 | Moderate |
| Ciampi (2014) | 2 | 2 | 0 | 1 | 0 | 2 | 1 | 0 | 2 | 2 | 2 | 1 | 15 | Moderate |
| Flury (2018) | 2 | 2 | 2 | 2 | 0 | 2 | 2 | 1 | 2 | 1 | 2 | 2 | 20 | Good |
| Kantanavar (2024) | 2 | 2 | 2 | 2 | 2 | 2 | 2 | 0 | 2 | 2 | 2 | 1 | 21 | Good |
| Maillot (2018) | 1 | 2 | 0 | 1 | 0 | 2 | 2 | 0 | 2 | 2 | 2 | 1 | 15 | Moderate |
| Rosales-Varo (2018) | 2 | 2 | 1 | 1 | 0 | 2 | 2 | 0 | 2 | 2 | 2 | 1 | 17 | Moderate |
| Ting (2023) | 2 | 2 | 2 | 2 | 1 | 1 | 2 | 1 | 2 | 1 | 2 | 1 | 19 | Moderate |
| Walton (2007) | 2 | 1 | 2 | 1 | 0 | 2 | 1 | 0 | 2 | 2 | 2 | 1 | 16 | Moderate |
| Yoon (2016) | 2 | 2 | 2 | 2 | 0 | 1 | 2 | 1 | 2 | 2 | 2 | 1 | 19 | Moderate |
| Zhang (2024) | 2 | 2 | 2 | 1 | 0 | 1 | 1 | 2 | 2 | 1 | 2 | 1 | 17 | Moderate |
