# Supplementary material for: Structural Augmentation in Rotator Cuff Repair Decreases the Risk of Retear: A Systematic Review and Meta-analysis
Source: Am J Sports Med. 2026 Jan 18;54(6):1525–36. doi: 10.1177/03635465251400356 (PMC13133422; doi:10.1177/03635465251400356)
Supplement: sj-docx-2-ajs-10.1177_03635465251400356 – Supplemental material for Structural Augmentation in Rotator Cuff Repair Decreases the Risk of Retear: A Systematic Review and Meta-analysis [file sj-docx-2-ajs-10.1177_03635465251400356.docx]

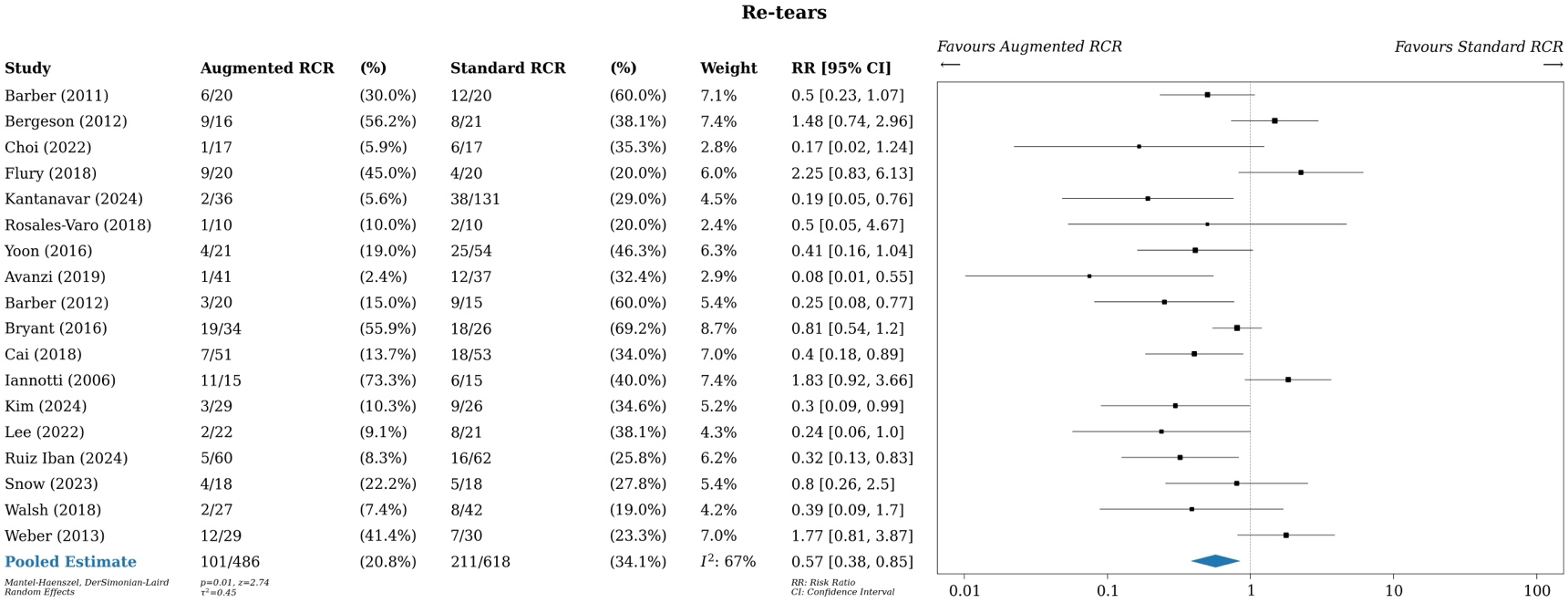


**Supplementary Figure 2.** Sensitivity analysis of re-tears including only studies evaluating primary RCR
